# Relationship between stroke onset timing and clinical outcomes in ischemic and hemorrhagic strokes: a systematic review

**DOI:** 10.1016/j.ijcrp.2025.200559

**Published:** 2025-12-14

**Authors:** Paria Heidari, Negar Heidari, Habibolah Khazaie, Sabra Margaret Abbott, Azad Maroufi, Amir Abdolmaleki, Nader Salari, Masoud Mohammadi, Amir Sharafkhaneh

**Affiliations:** a Student Research Committee, Kermanshah University of Medical Sciences, Kermanshah, Iran; b Social Development and Health Promotion Research Center, Kermanshah University of Medical Sciences, Kermanshah, Iran; c Sleep Disorders Research Center, Kermanshah University of Medical Sciences, Kermanshah, Iran; d Northwestern Feinberg, School of Medicine, Chicago, IL, USA; e Neurosciences Research Center, Research Institute for Health Development, Kurdistan University of Medical Sciences, Sanandaj, Iran; f Department of Operating Room, Nahavand School of Allied Medical Sciences, Hamadan University of Medical Sciences, Hamadan, Iran; g Fertility and Infertility Research Center, Health Technology Institute, Kermanshah University of Medical Sciences, Kermanshah, Iran; h Research Center for Social Determinants of Health, Jahrom University of Medical Sciences, Jahrom, Iran; i Department of Medicine, Pulmonary, Critical Medicine and Sleep Medicine, Baylor College of Medicine, Houston, TX, USA

**Keywords:** Stroke outcomes, Ischemic stroke, Hemorrhagic stroke, Systematic review

## Abstract

**Background:**

Cerebrovascular accident (CVA) is a critical medical pathology caused by the interruption of cerebral blood flow and leads to neuronal injury or neurodegeneration. Generally, CVAs are classified into two primary categories of ischemic and hemorrhagic. Investigation of circadian rhythm variation at the time of CVA onset is a critical subject for patient management, clinical treatment, and further scientific research. This systematic review aimed to investigate the relationship between the timing of stroke onset and patient outcomes in both ischemic and hemorrhagic strokes.

**Methods:**

Following searching strategy designation, international databases of Embase, PubMed, Scopus, WoS, ScienceDirect, and Google Scholar were searched using the MeSH-based keywords. No time restrictions were applied in this regard (by December 8, 2024). All English-based observational studies reporting the risk of CVA at various daytimes were enrolled for screenings and quality control. Finally, eligible studies were selected for data extraction and categorization.

**Results:**

According to the reports, ischemic strokes strictly occur in morning hours (06:00 to 12:00) while some studies recorded a bimodal pattern (dual peaks during morning and evening). It was also established that the strokes at night were more severe and yielded more unfavorable results. Scattered reports were found regarding the hemorrhagic strokes; some papers revealed the highest incidence in the early morning hours, while others reported the late evening or nighttime occurrence. In addition, some studies showed that nighttime hemorrhagic strokes are usually associated with greater severity and unfavorable clinical outcomes.

**Conclusion:**

This study clarified the effect of cyclic pattern on the incidence and outcome of stroke. These trends seem greatly accounted for improvement of prevention strategies, management of treatments, and enhancement of patient outcomes.

## Highlights

• Searching in Embase, PubMed, Scopus, WoS, ScienceDirect, and Google Scholar.
• No time restrictions were applied in this regard (by December 8, 2024).
• The findings indicated that ischemic strokes overwhelmingly occurred in the morning hours (06:00 to 12:00).
• This clearly states the effect of cyclic pattern on the incidence and outcome of stroke.

## 1 Background

Cerebrovascular accident (CVA) is a critical medical condition caused by the interruption of cerebral blood flow and severe neurodegeneration. Generally, Cerebrovascular accident (CVA) is categorized into two primary types of ischemic and hemorrhagic [1,2]. Ischemic Cerebrovascular accident (CVA) is most prevalent, accounting for approximately 87 % of CVA cases [3], and occurs due to occlusion of the cerebral blood vessels, typically by thrombosis or plaque accumulation [4]. Conversely, the hemorrhagic Cerebrovascular accident (CVA) (20–30 % of cases) leads to the rupture and intracranial hemorrhage [2,5].

Multiple influential factors are associated with the occurrence of various types of CVAs. For instance, hypertension and alcoholism are considerably correlated with hemorrhagic CVA, whereas dyslipidemia is more closely related to ischemic Cerebrovascular accident (CVA) [4]. Diabetes mellitus can also increase the risk of both types of CVA; type I diabetes is associated with a 4-fold increased risk of hemorrhagic CVA [6]. Age and gender also play a critical role in CVA onset. Generally, the risk of both ischemic and hemorrhagic CVAs increases with age, and males represent approximately 20 % higher propensity to hemorrhagic type than females. This difference may be attributed to the protective effects of estrogen in women, particularly prior to menopause [7].

Different studies clarify that the timing of CAV onset has a great deal of difference. The highest incidence of CVAs occurs mostly in the morning, between 6:00 and 12:00 [[8], [9], [10]]. This diurnal rhythm is expected due to the fluctuations in autonomic nervous activity as well as variations in physiological status, such as blood pressure and coagulation [11]. Likewise, there are sleep-disordered changes in the circadian rhythm, physical activity, and wake-sleep patterns, which possibly trigger the CVA [12]. Variations in the timing of cerebrovascular accident (CVA) onset have substantial implications for clinical management. In particular, the patients awaken with neurological deficits often present an indeterminate symptom onset time with significant challenges for selection and initiation of appropriate therapeutic interventions [13].

Researches indicate that nocturnal or early morning CVAs are typically associated with more dangerous symptoms and poor functional outcomes, potentially due to delays in hospital arrival and no appropriate treatment [14]. Conversely, CVAs' onset in the evening generally presents milder symptoms [15]. Thus, understanding the circadian patterns of CVA onset may enhance clinical management and outcomes.

According to a study, a 28-day mortality index following CVA onset was higher in the morning [16], and another study reported similar findings for CVA onset at various daytimes [17]. In addition, the daily fluctuations of CVA are critical factors in patient management, particularly regarding thrombolytic therapy. These patients develop stroke symptoms during the night, yet frequently fail to receive timely intervention, as the onset is unwitnessed and information from relatives regarding their neurological status is often insufficient for precise clinical decision-making [18].

Numerous studies investigated the relationship between circadian rhythm and CVA onset. A meta-analysis with 11,816 CVA patients [19] reported that during ischemic, hemorrhagic, and even transient attacks, the risk of CVA is significantly higher during the morning hours, particularly between 6:00 a.m. and 12:00 noon. Specifically, the risk of ischemic CVA is increased by 89 %, hemorrhagic type by 52 %, and transient ischemic attack by 80 % compared to the remaining 18 h of daytime. Furthermore, the risk of nocturnal CVA (between midnight to 6) is significantly lower for all types of CVA: 30 % for ischemic, 54 % for hemorrhagic, and 81 % for transient ischemic attacks compared to the remaining 18 h of daytime [19].

Since there are scattered reports regarding the CVA, this study was designed to examine the relationship between the timing of stroke onset and the prognosis of ischemic and hemorrhagic strokes, with the aim of enhancing clinical management and designation of preventive strategies for stroke regarding public health consequences.

## 2 Methods

### 2.1 PRISMA guidelines

Systematic searching was applied based on PRISMA guidelines [20], including Topic selection, Searching strategy designation, Primary and secondary screenings, Study selection, Inclusion and exclusion criteria implementation, Qualitative assessment, and Data collection and categorization. Full texts of the retrieved articles were independently reviewed by two authors. To prevent any probable bias, all stages of source reviewing and data extraction were applied independently by two researchers. In the case of disagreement between both researchers, the article was reviewed by a third person.

### 2.2 MeSH keywords and searching strategy

In this regard, international databases of Embase, PubMed, Scopus, WoS, ScienceDirect, and Google Scholar were systematically searched by December 8, 2024. Searching was applied using MESH terms of *“Circadian variation”, “CVA”, “Cerebrovascular accident”, “Transient ischemic attack”,* and *“Brain attack”*. PubMed sample search was also designed as; (((((((Stroke[Title/Abstract]) OR (Cerebrovascular Accident[Title/Abstract])) OR (CVA[Title/Abstract])) OR (Cerebral Stroke[Title/Abstract])) OR (Cerebrovascular Accident, Acute[Title/Abstract])) OR (Cerebrovascular Stroke[Title/Abstract])) AND (Transient ischemic attack[Title/Abstract])) OR (Ischemic Attack, Transient[Title/Abstract])) OR (Anterior Circulation Transient Ischemic Attack[Title/Abstract])) OR (Brain Stem Transient Ischemic Attack[Title/Abstract])) OR (Brainstem Ischemia, Transient[Title/Abstract])) OR (Brainstem Transient Ischemic Attack[Title/Abstract])) AND (Brain attack[Title/Abstract])) AND (Circadian variation[Title/Abstract])) OR (Circadian Rhythm[Title/Abstract])))))))

### 2.3 Inclusion and exclusion criteria

All English-based observational studies reporting the risk of CVA across various daytimes with available full text and extractable data were totally included in this study. Besides, animal, experimental, interventional, and case studies, investigations with unavailable full texts, non-English papers, duplicates, articles with insufficient data, and Letters to the editor were excluded.

### 2.4 Quality assessment

The Newcastle-Ottawa Scale is a quality assessment tool for observational studies. The NOS assigns up to a maximum of nine points for the least risk of bias in three domains of Selection of study groups (4 points), Comparability of groups (2 points), and Ascertainment of exposure and outcomes (3 points) for case-control and cohort studies, respectively, and 11 scores are possible. Eventually, articles were classified as high quality (scoring ≥5 points) or low quality (scoring<5 points).

### 2.5 Data extraction

Independently, two authors reviewed the eligible studies based on inclusion and exclusion criteria. Initially, Titles and Abstracts of the articles were examined, irrelevant and duplicate studies were excluded, and subsequently, full texts of the articles were reviewed for further assessments. Following the selection of eligible papers, the main data were extracted, including “First author's name”, “CVA subtype”, “Time of CVA”, “Time intervals”, and “Outcomes” (Table 1).

**Table 1:** Findings from studies on circadian rhythm variations in the timing of stroke onset. Early neurological deterioration, mini-mental state examination, metabolic equivalent of task, cerebrovascular disease, cerebral infarction, subarachnoid hemorrhage, intracerebral hemorrhage, thromboembolic cerebral infarction, hemispheric infarct ratio.

Author	Year	symptom onset time/ medical presentation time	Type of CVA	Time Blocks (h), starting at	n	00:00–05:59	06:00–11:59	12:00–17:59	18:00–23:59	Untimed	Findings
Albarracín et al. [33]	2024	symptom onset time	Ischemic	4, MN	1008	57	296	354	301	581	The majority of patients, regardless of CVA type, were distributed during the hours of 08:00 to 12:00 and 12:00 to 16:00. Significant differences in temporal distribution based on CVA type were observed; intracerebral hemorrhage (ICH) was more frequently noted during the period of 04:00 to 08:00, with a majority of these patients having a history of hypertension. Transient ischemic attack (TIA) was predominantly observed during the period of 12:00 to 16:00, while CVA mimics were more prevalent at 20:00 (8 p.m.). Ischemic CVA was uniformly distributed across various time periods throughout the day. The seasonal pattern of CVA incidence showed no differences among various types of CVA, with winter recording the highest number of cases.
			Intracerebral hemorrhage		194	14	67	48	65	106	
			Transient ischemic attack		77	2	23	35	17	12	
			Mimic		358	12	94	122	130	12	
Rahman et al. [45]	2024	symptom onset time	Ischemic, Hemorrhagic, and Subarachnoid Hemorrhage	6, MN	100	19	46	24	11	0	Hypertension is recognized as a significant risk factor in 72 % of cases. CVA symptoms predominantly manifest between 06:01 and 12:00 (46 %).
Sadhukhan et al. [31]	2024	symptom onset time	Any strokes	4, MN	249	47	76	67	59	0	Patients with nocturnal onset of cerebrovascular accidents exhibited a higher National Institutes of Health Stroke Scale (NIHSS) score (12.2 ± 5.67) upon admission compared to other subgroups (8.73 ± 5.92). In the same cohort (nocturnal onset CVA), a greater number of individuals with diabetes mellitus were observed, presenting with elevated fasting blood glucose levels (186.57 ± 93.40). After 6 months, individuals whose CVA commenced at night demonstrated a higher prevalence of language disorders and elevated depression scores. A significant reduction in the mRNA levels of BMAL1 and CRY1 genes was observed, correlating with raw scores for language disorders and depression; data pertaining to nocturnal onset CVA corroborated these findings. However, the higher incidence of CVA during daytime did not indicate any genetic correlation.
Ryu et al. [30]	2022	symptom onset time	Ischemic	12,4 a.m.	10757	3900	3801	2220	836	3729	12.7 % of patients experienced adverse outcomes (END), indicating a deterioration in their condition. Nocturnal CVAs were associated with a higher incidence of adverse outcomes and had a lower likelihood of achieving a favorable result (good functional outcome). The National Institutes of Health Stroke Scale (NIHSS) score was elevated in patients with CVA onset during nighttime. At three months post-stroke, patients with morning CVA (06:00 to 10:00) demonstrated superior functional outcomes compared to those whose CVAs occurred at night.
Wang et al. [32]	2022	symptom onset time	Any stroke	6, MN	438	58	151	134	95	0	The results of this study indicated that 58.6 % of patients whose cerebrovascular accident occurred between 00:00 and 06:00 achieved a favorable outcome (good performance). This proportion was 43.7 %, 36.6 %, and 30.5 % for the other groups, respectively. Patients with CVA onset during the hours of 00:00 to 06:00 and 06:00 to 12:00 were more likely to achieve a favorable outcome compared to patients whose CVA onset occurred between 18:00 and 24:00. The timing of CVA onset had no impact on the mortality rate at 3 months, symptomatic intracerebral hemorrhage, or early success in the restoration of blood flow.
Fodor et al. [41]	2020	symptom onset time	Ischemic	6, MN	63	5	34	16	8	0	The study indicates that the temporal pattern of CVA onset is corroborated by a recognized pattern demonstrating an increased incidence of ischemic CVA in the morning. Over time, the Mini-Mental State Examination (MMSE) score, utilized for assessing cognitive function, exhibits varying changes depending on the timing of CVA occurrence. Patients whose CVA commenced at night appear to demonstrate lesser cognitive advancement in the second year following ischemic CVA compared to patients whose CVA occurred during other hours of the day.
Al-Ahwal et al. [46]	2019	symptom onset time	Any strokes	6, MN	98	16	36	27	19	0	Both types of CVA, namely ischemic CVA and hemorrhagic CVA, exhibit a distinct temporal pattern in onset, characterized by a peak occurrence in the morning and a minimal occurrence at night. Following a CVA, the natural fluctuations in blood pressure throughout the diurnal cycle (circadian blood pressure pattern) are abnormally diminished or abolished.
Kabir et al. [21]	2019	symptom onset time	Ischemic	6, MN	40	13	19	6	2	0	The highest incidence of cerebrovascular accidents occurred between 6:00 a.m. and 12:00 p.m. (47.8 %), followed by 12:00 a.m. to 6:00 a.m. (25.4 %), 12:00 p.m. to 6:00 p.m. (17.9 %), and 6:00 p.m. to 12:00 a.m. (9 %). Circadian variations in CVA occurrence among different age groups (under 65 years and 65 years or older) as well as in relation to sex, smoking habits, presence or absence of diabetes mellitus, atrial fibrillation, and dyslipidemia were homogeneous and statistically nonsignificant. However, hypertension and ischemic heart disease (IHD) were significantly associated with circadian variations in CVA occurrence. The occurrence of ischemic and hemorrhagic CVA was higher between 6:00 a.m. and 12:00 p.m. (47.5 % and 48.1 %, respectively). Additionally, significant circadian variations were observed for both types of CVA (ischemic and hemorrhagic).
			Hemorrhagic		27	4	13	6	4	0	
Lee et al. [35]	2019	symptom onset time	Aneurysmal Subarachnoid Hemorrhage	4, MN	234	25	92	61	56	101	The incidence of cerebrovascular accidents exhibits a distinct bimodal pattern, peaking between 08:00 and 12:00 h and again from 16:00 to 20:00 h. This finding is statistically significant (p < 0.001). The metabolic equivalent of task (MET) values (a measure of physical activity) were significantly higher during all examined time intervals compared to nocturnal values (00:00 to 04:00 h) (p < 0.031). The distribution of MET values demonstrates a uniform pattern that slightly differs from the bimodal pattern of CVA onset timing. No significant differences in odds ratios for any time interval based on MET levels were observed.
Choi et al. [22]	2015	symptom onset time	Ischemic	6, MN	968	104	363	299	202	518	CVAs are categorized by season as follows: spring (26.5 %), summer (27.2 %), autumn (24.1 %), and winter (22.3 %). Ischemic CVA (a type of cerebrovascular accident) exhibits significant variations in onset timing, with meaningful fluctuations observed throughout the day (p < 0.001). The highest incidence of CVA occurs between 6:01 a.m. and 12:00 p.m. (37 %), and this pattern is consistent across all seasons.
Raj et al. [23]	2015	symptom onset time	Ischemic	6, MN	247	73	100	58	16	216	The highest incidence rate of cerebrovascular accidents has been observed in the late hours from 06:00 to 11:59, with this difference being statistically significant compared to other times of the day (P < 0.001). This pattern is consistent for both types of CVA, namely ischemic and hemorrhagic. The lowest incidence rate of cerebrovascular accidents has been noted in the late hours from 18:00 to 23:59. Although there was no statistically significant difference in the occurrence of CVA between the two six-month periods, an increasing trend in the number of CVA patients was observed from November to February. No differences in the occurrence of CVA were noted between different types of CVA (ischemic and hemorrhagic) or within each type across various seasons with differing temperatures. The occurrence of ischemic CVA is not dependent on a specific time or season.
			Hemorrhagic		120	29	43	32	16		
Fodor et al. [47]	2014	symptom onset time	Ischemic	6, MN	969	64	440	161	155	149	Three types of CVA indicate daily variations in their occurrence. This means that the highest incidence of these CVAs is observed in the morning, while the lowest incidence occurs at night. This daily pattern is independent of demographic factors (such as age and sex) and vascular risk factors (such as hypertension or diabetes mellitus). In other words, these daily variations in CVA occurrence are not influenced by these factors.
			Hemorrhagic		94	8	52	16	12	6	
			Subarachnoid Hemorrhage		20	2	8	4	3	3	
Hossain et al. [48]	2014	symptom onset time	Any strokes	4, MN	402	141	129	78	54	0	The highest incidence of cerebrovascular accidents (26.9 percent) was recorded between 4:01 a.m. and 8:00 a.m., while the lowest incidence (7.5 percent) occurred between 8:01 p.m. and 12:00 a.m. The timing of cerebrovascular accidents is influenced by the sleep-wake cycle, with the greatest risk of occurrence during the early morning hours.
Koo et al. [49]	2014	symptom onset time	Any strokes	6, MN	2047	189	664	661	533	179	A significant diurnal rhythm in the incidence of cerebrovascular accidents based on the onset time of symptoms has been observed (p < 0.001). The peak occurrence of CVA was recorded at 12:22. The results indicated significant diurnal variations in age, fasting blood glucose, high-density lipoprotein (HDL), and low-density lipoprotein (LDL). The daily distribution of HDL was found to be similar to the distribution of CVA occurrences.
Kumar et al. [50]	2014	symptom onset time	Ischemic	6, MN	181	36	81	45	19	0	A majority of patients (44.8 percent) presented with acute ischemic CVA that occurred between the time frame of 6:00 to 12:00 h. The mean age of the patients was 69.09 years, with ages ranging from 36 to 97 years. A majority of the patients (74.6 percent) were male.
Temes et al. [51]	2012	symptom onset time	Aneurysmal Subarachnoid Hemorrhage	6, MN	251	38	98	58	57	8	The peak incidence of aneurysmal subarachnoid hemorrhage (aSAH) occurred between 07:00 and 08:00 h, comprising 13 % of the sample. Additionally, a significant phase of occurrence was recorded at 7.33 h. The incidence of aSAH in the morning was significantly higher than that during the night. Individuals who were non-smokers were more likely to experience aSAH in the morning compared to those who were smokers.
Naess et al. [29]	2011	symptom onset time	Cerebral infarction	6, MN	1101	658	144	95	202	0	Lacunar CVA (a subtype of ischemic CVA) predominantly occurs during nighttime hours, particularly between midnight and 6 a.m.
Turin et al. [44]	2010	symptom onset time	Hemorrhagic	6, MN	429	51	155	133	90	70	The proportion of hemorrhagic CVA is highest in the morning and lowest at night. An increase in the incidence of CVA in the morning has been observed in both genders, in individuals under 65 years and over 65 years, as well as in both types of hemorrhagic CVA: intracerebral hemorrhage and subarachnoid hemorrhage. The elevated risk of CVA during the daytime persisted even after adjusting for age, gender, and other risk factors.
Carcel et al. [34]	2009	symptom onset time	Ischemic	6, MN	96	13	55	17	11	25	Ischemic CVA exhibits the highest incidence between 06:00 and 12:00. Hemorrhagic CVA predominantly occurs between 18:01 and 23:59 (18:00 to midnight). Ischemic heart disease and hypertension are recognized as significant clinical risk factors for CVA occurrence.
			Hemorrhagic		44	8	10	15	11		
Turin et al. [25]	2009	symptom onset time	Ischemic	6, MN	637	89	259	168	120	62	The incidence of these CVAs is highest in the morning and lowest at night. In the morning, the occurrence of ischemic CVA is more frequently observed in both genders, in individuals under 65 years and over 65 years, and across all subgroups of ischemic CVA. The increase in the occurrence of ischemic CVA in the morning is consistent across all seasons and days of the week. This trend persists even after adjustment for age, gender, and other risk factors.
Yun et al. [52]	2007	symptom onset time	Ischemic	6, MN	274	57	89	80	48	12	The highest incidence of ischemic CVA occurred between 06:01 and 12:00 (89 patients, 30.2 %). The lowest risk of ischemic CVA was observed between 18:01 and 24:00 (48 patients, 16.3 %).
			Hemorrhagic		21	2	9	1	9		
Omama et al. [27]	2006	symptom onset time	Cerebral Infarction	2, MN	5060	643	2179	1241	997	1727	CVA Onset Pattern CIF: In all onset conditions, daily variations indicate a sharp peak in the morning (from 06:00 to 07:59) and a smaller peak in the afternoon (from 18:00 to 19:59). Additionally, there is a slight decline around noon and a minimum during the night. During wakefulness, two peaks are observed: one from 10:00 to 11:59 and another from 18:00 to 19:59. CVA Onset Pattern ICH and SAH: These two types of CVA also exhibit two similar peaks in the morning and afternoon. During sleep, a peak is observed in the morning (from 06:00 to 07:59).
			Intracerebral Hemorrhage		3159	306	1073	900	880	460	
			Subarachnoid Hemorrhage		1308	166	420	368	354	148	
GUPTA et al. [26]	2005	symptom onset time	Ischemic	6, MN	100	24	47	18	11	15	The distribution of age and sex at four different time points of CVA onset indicated that there was no significant difference between the groups (p < 0.2). Among 117 patients, the peak onset of ischemic CVA occurred in 47 patients (47 percent) between the hours of 6:01 and 12:00. The lowest risk for ischemic CVA onset was observed between the hours of 18:01 and 24:00. The distribution of vascular risk factors at various times of CVA onset demonstrated that patients with ischemic heart disease (IHD) had a higher likelihood of experiencing a CVA between the hours of 6:01 and 12:00 (p < 0.05). Additionally, the presence of hypertension showed a significant association during late morning hours (p < 0.01). Regression analysis revealed that hypertension was the most significant risk factor associated with ischemic CVA onset during late morning hours (p < 0.01).
			Hemorrhagic		17	4	5	5	3		
Kocer et al. [37]	2005	symptom onset time	Any strokes	6,3 a.m.	1199	468	280	272	179	0	The highest incidence of cerebrovascular accidents (32.4 %) occurred between 03:00 and 06:00 h, with this time associated with a 32.4 % increase in relative risk of CVA compared to other times. Additionally, the lowest risk of CVA was observed during the hours between 21:00 and 23:59. Seasonal variations do not impact the prevalence of cardiovascular disease (CVD); however, ischemic CVA (IS) is more frequently observed in summer, while intracerebral hemorrhages (ICH) and subarachnoid hemorrhages (SAH) are more prevalent in winter. The presence of hypertension at a specific time of day, specifically between 00:01 and 03:00, exhibited the highest frequency. Notably, 64.5 % of patients with cerebrovascular accidents had hypertension during this time frame. However, the relationship between the timing of CVA onset and blood pressure or other modifiable risk factors (such as diabetes mellitus, obesity, and lifestyle) was not statistically significant. This indicates that while hypertension was more frequently observed during this specific time, it cannot be considered a definitive factor for CVA occurrence on its own.
Serena et al. [53]	2003	symptom onset time	Any strokes	6, MN	1248	231	389	303	325	0	The peak incidence of cerebrovascular accidents has been observed between 6:01 a.m. and 12:00 p.m.
Spengos et al. [28]	2003	symptom onset time	Any strokes	2, MN	1120	155	441	309	215	133	The highest incidence of cerebrovascular accidents occurs in the morning hours (between 06:00 and 12:00), with a peak incidence (between 08:00 and 10:00). Additionally, a second peak with lower frequency has been observed in the late afternoon (between 16:00 and 18:00). The number of CVA occurring during these times has been significantly higher than the expected number. Analysis of CVA onset timing for various CVA subgroups also indicates a similar bimodal distribution, with the first peak and highest incidence occurring between 08:00 and 10:00 and the second peak between 16:00 and 18:00 for most subgroups, except for lacunar CVA, which occurs more frequently during the night.
Bhalla et al. [54]	2002	symptom onset time	Any strokes	4, MN	146	31	39	46	30	0	The highest number of events was observed in the time interval between 4:00 a.m. and 8:00 a.m. (34 out of 146) as well as between 12:00 p.m. and 4:00 p.m. (34 out of 146). The lowest number of events was recorded in the time interval between 12:00 a.m. and 4:00 a.m. (14 out of 146).
Gur et al. [24]	2000	symptom onset time	Ischemic	4, MN	1769	169	814	477	309	0	The peak incidence of cerebrovascular accidents (599 patients, 34 percent) occurred between 06:00 and 10:00 h. The distribution of age and sex for different onset times of CVA has also been examined. The age of patients did not show a statistically significant difference (P < 0.2), but the male sex was significantly more prevalent in CVA that occurred between 22:00 and 02:00 (P < 0.05). The distribution of vascular risk factors for different onset times of CVA was also investigated, revealing no statistically significant differences among common vascular risk factors for morning CVA compared to those occurring at other times. Patients with hypertension and ischemic heart disease had a higher likelihood of experiencing a CVA between 22:00 and 02:00 (P < 0.05). The distribution of vascular territory, recurrence, and severity of ischemic CVA for different onset times was also analyzed, with none of these parameters showing a statistically significant increase for morning CVA (P < 0.2).
Bassetti et al. [55]	1999	symptom onset time	Transient ischaemic attack and Ischaemic	6, MN	109	23	46	26	14	0	Patients with cerebrovascular accidents occurring during daytime and nighttime were similar in terms of demographics (age, sex, etc.), risk factors (such as hypertension, diabetes mellitus, etc.), associated vascular diseases, clinical characteristics, sleep features (such as severity of sleep apnea), and CVA severity, cause, and outcome. The only significantly different variable was diastolic blood pressure at the time of admission. Patients with nocturnal CVA exhibited lower diastolic blood pressure (74 versus 82 mmHg, p = 0.01).
Chaturvedi et al. [56]	1998	symptom onset time	Atrial Thrombus	6, MN	173	22	59	49	43	0	Timing of Occurrence of Different Types of CVA: Atherothrombotic CVA: The highest percentage of this type of CVA occurred between 6:01 a.m. and 12:00 p.m. Cardioembolic CVA: The highest percentage of this type of CVA also occurred within the same time frame (6:01 a.m. to 12:00 p.m.). CVA with other or unknown mechanisms: This type of CVA also exhibited the highest incidence during the same time period. Lacunar CVA: The highest occurrence of this type of CVA was noted upon awakening. More than half of the CVAs in this study occurred either upon awakening or during the mid to late morning hours. The association between CVA type and the onset of symptoms was not statistically significant (P = 0.07).
			Cerebral Embolism		210	18	81	65	46	0	
			Lacunar Stroke		210	12	69	77	52	0	
			Other/ Unknown		356	35	127	101	93	0	
Lago et al. [57]	1998	symptom onset time	Ischemic	6,2 a.m.	914	101	395	247	171	309	The results indicate that the highest incidence of CVA occurs between 6:01 a.m. and noon, while the lowest incidence is observed at night. No significant differences were observed in age, sex, blood pressure, diabetes mellitus, and ischemic cardiomyopathy based on the time of CVA onset and type of CVA. There was no difference in the timing of CVA onset between patients with first-ever CVA and those with recurrent CVA.
Vermeer et al. [42]	1997	symptom onset time	Subarachnoid hemorrhage	2, MN	258	31	72	84	71	27	The risk of aneurysm rupture during the night (from midnight to 6 a.m.) in this study, as well as in the combined dataset, was lower. In other words, during this period, the probability of aneurysm rupture is reduced. The risk of aneurysm rupture during the day and evening remains elevated, reaching its minimum around noon (near 12 o'clock). This indicates that during the daytime, the likelihood of aneurysm rupture is increased. For peri-mesencephalic hemorrhage, similar fluctuations in onset time were observed, but without reaching a minimum at noon. This signifies that in this type of hemorrhage, the pattern of temporal fluctuations differs from that of aneurysm rupture.
Hayashi et al. [58]	1996	symptom onset time	Fatal	2, MN	529	93	141	120	175	15	Between the hours of 08:00 and 10:00, the incidence of fatal cerebrovascular accidents significantly increases. There is another peak between the hours of 18:00 and 20:00, during which the occurrence of cerebrovascular accidents also rises. The incidence of cerebrovascular accidents in the afternoon and evening (between 18:00 and 20:00) is significantly higher than that observed in the early morning hours (between 00:00 and 02:00). Diurnal variations in both males and females were generally similar, indicating that both sexes experienced fatal cerebrovascular accidents at comparable times.
Haapaniemi et al. [59]	1996	symptom onset time	Ischemic	6, 2 a.m.	610	170	189	149	102	113	Among young adults (16–40 years) and females, a higher incidence of cerebrovascular accidents occurred during weekends and holidays than anticipated. Notably, young females faced an increased risk of CVA during these periods. Factors associated with the occurrence of cerebrovascular accidents on weekends and holidays included age 16–30 years, female gender, and alcohol consumption close to the time of the CVA, whereas smoking on weekdays was linked to CVA occurrence. Among middle-aged individuals, an increase in the incidence of cerebrovascular accidents was observed in the morning (on weekends, holidays, and weekdays); however, among young adults, an increase in CVA incidence in the evening was also noted during weekends and holidays as well as on weekdays.
Kelly-Hayes et al. [36]	1995	symptom onset time	Any strokes	4, MN	401	71	180	100	50	234	Winter was identified as the peak season for the occurrence of embolic CVA. The highest incidence of CVA occurred on Mondays, particularly among employed males. For intracerebral hemorrhages, one-third of these CVAs occurred on Mondays in both genders. The times when CVAs were most prevalent were between 8 a.m. and noon. This pattern was consistent across all subtypes of CVA, and even when individuals who experienced CVA while asleep or upon waking were excluded from the study, this pattern remained evident.
Roberts et al. [60]	1994	symptom onset time	Any strokes	1, MN	68	10	33	14	11	6	The pattern of CVA occurrences throughout the day indicates a significant increase (P < 0.001) in the frequency of CVA beginning at 6:00 a.m., peaking between 7:00 and 8:00 a.m., and subsequently decreasing until 10:00 a.m., with no other significant differences observed throughout the day. No significant differences in CVA timing were observed between male and female patients. When comparing patients with hypertension (39 patients) to those without (22 patients), no significant differences in CVA timing were observed between the two groups.
Pardiwalla et al. [61]	1993	symptom onset time	Any strokes	8, 6 a.m.	182	26	82	46	28	0	The incidence of cerebrovascular accidents in patients demonstrated the highest frequency between 6:01 a.m. and 2:00 p.m., encompassing individuals with both ischemic and hemorrhagic CVA. Patients with hypertension also exhibited similar temporal patterns in the occurrence of cerebrovascular accidents. Identifying high-risk periods may assist in tailoring medication dosages to times of vulnerability.
Gallerani et al. [62]	1993	symptom onset time	Any strokes	6, MN	897	107	330	276	184	80	The majority of CVAs occurred in the morning between 7:00 a.m. and noon (35 %), and the hypothesis of uniform distribution of onset time was rejected. A circadian rhythm for ischemic CVA and transient ischemic attacks was identified, with peaks at 11:56 a.m. and 12:41 p.m., respectively. An annual periodicity for ischemic CVA was also identified, with a predominant peak in October. The spectral analysis identified a circadian cycle for intracerebral hemorrhage with a period of 4 h and an annual cycle for transient ischemic attacks with a period of 4 months.
Haapaniemi et al. [59]	1992	symptom onset time	First ischemic	2, MN	609	101	277	131	100	0	The timing of cerebrovascular accidents significantly differs between weekdays and holidays (including Saturday and Sunday) (p < 0.001). The incidence of ischemic CVA peaks on weekdays between 6:00 and 8:00 a.m. and on holidays between 8:00 and 10:00 a.m. CVA occurs more frequently within 2 h after awakening than at any other time of the day. In individuals of working age, the occurrence of ischemic cerebrovascular accidents increases in the early morning, and this pattern varies depending on the type of day (weekday or holiday).
Wroe et al. [63]	1992	symptom onset time	Any first stroke	2, MN	554	59	265	137	93	80	For all types of CVA, there is a significant daily variation (p < 0.001) with a peak incidence occurring between 08:00 and 10:00 h. This pattern persists even after accounting for CVA identified during wakefulness (p < 0.001). Related
Franke et al. [64]	1992	symptom onset time	Intracerebral Hemorrhage	6, MN	138	21	37	52	28	19	Intracerebral hemorrhage occurs more frequently during the daytime (from 6 a.m. to 6 p.m.) compared to nighttime and evening (p < 0.01). No seasonal variation in the incidence of intracerebral hemorrhage was observed, neither in the overall patient population nor in subgroups with a history of hypertension or those aged less than or greater than 65 years. Hypertension may contribute to the occurrence of intracerebral hemorrhage; however, its presence is not essential for the occurrence of hemorrhage.
Ricci et al. [65]	1992	symptom onset time	Any strokes	3, MN	368	32	180	105	51	7	Cerebrovascular accidents (48 %), primary intracerebral hemorrhages (54 %), subarachnoid hemorrhages (53 %), and "undetermined" CVA (51 %) predominantly occur between the hours of 6 a.m. and noon. This peak incidence of CVA persists even when CVAs identified during wakefulness are uniformly distributed throughout the sleep period. Within the subgroup of cerebrovascular accidents, lacunar syndromes (a type of CVA) are more frequently observed during sleep. Cerebrovascular accidents occur more commonly in winter, while primary intracerebral hemorrhages are more prevalent in autumn.
Sloan et al. [66]	1992	symptom onset time	Intracerebral and Subarachnoid Hemorrhage	2, MN	375	32	134	124	85	105	52.5 % of patients reported the onset time of intracerebral hemorrhage between 06:00 and 14:00. The peak occurrence of this type of hemorrhage was observed between 10:00 and 12:00 (p < 0.001), indicating a distinct temporal pattern for the occurrence of this type of CVA. Patients with subarachnoid hemorrhage had a significantly lower history of hypertension compared to those with intracerebral hemorrhage (p < 0.001). The onset time of subarachnoid hemorrhage during the day was more uniform for patients, although this difference was not entirely statistically significant (p = 0.074). Patients experiencing subarachnoid hemorrhage with a history of hypertension were more likely to have their peak occurrence time in the mid to late morning compared to patients without a history of hypertension (p < 0.001). Additionally, variability in the onset time of intracerebral hemorrhage persisted, even when patients with indeterminate onset times were randomly considered between midnight and 08:00. In this study, no distinct seasonal patterns were observed, nor was there a relationship between initial systolic or diastolic blood pressure and onset time for either type of hemorrhage.
Toni et al. [67]	1991	symptom onset time	Ischemic	1, MN	80	1	36	18	25	0	45 % of cerebrovascular accident cases have occurred between 6:01 a.m. and noon. 22.5 % of cases have occurred between noon and 6 p.m. 31.25 % have occurred between 6:01 p.m. and midnight. Only 1.25 % of cases have occurred between midnight and 6 a.m. These results are statistically significant (p < 0.0001). Activation of the catecholaminergic system in the morning may explain this pattern of ischemic CVA onset. In other words, the hormonal and physiological changes that occur in the morning may influence the incidence of cerebrovascular accidents.
Herderscheê et al. [68]	1991	symptom onset time	Any strokes	6, MN	85	13	31	27	14	35	The interval between the onset of neurological deficits (CVA symptoms) and the initiation of treatment may be a critical factor in the management of acute cerebrovascular accidents. Delays in admission are categorized into two types: patient delay and medical delay. Multiple regression analysis was conducted with various factors such as CVA type (hemorrhagic or ischemic), age, history of CVA, etc. Only the variable of symptom onset time (from 6:01 a.m. to 12:00 p.m.) significantly reduced total delay and patient delay (p < 0.05).
Manfredini et al. [69]	1990	symptom onset time	Any strokes	1?∗, MN?	108	16	22	38	32	33	The results indicate a seasonal pattern in the onset of disease, characterized by an annual rhythm, with a peak occurring from late winter to early spring. A significant diurnal rhythm was also identified, with a peak in the afternoon.
Argentino et al. [70]	1990	symptom onset time	Ischemic	1, MN	426	66	239	86	35	0	335 patients were awake at the time of CVA onset (symptom onset between 6:01 a.m. and 11 p.m.), and 91 patients awoke with CVA symptoms (symptom onset between 11:01 p.m. and 8 a.m.). 56.1 % of CVA (239 cases) occurred between 6:01 a.m. and noon, with a peak hourly incidence between 7:01 and 8 a.m. (70 cases). 20.2 % (86 cases) occurred between 12:01 p.m. and 6 p.m., 8.2 % (35 cases) from 6:01 p.m. to midnight, and 15.5 % (66 cases) from 12:01 a.m. to 6 a.m. To assess the impact of CVA risk factors on circadian rhythm, subgroups of patients who smoked (130 individuals), had hypertension (217 individuals), or had diabetes mellitus (65 individuals) were analyzed. For these subgroups, the circadian variations of CVA were similar to those observed in the overall population. A similar pattern was also observed for the subgroup of patients with atrial fibrillation (54 individuals).
Marsh et al. [71]	1990	symptom onset time	Ischemic	2, MN	151	20	86	21	24	0	The onset time of ischemic CVA across all subgroups (such as small vessel CVA, cardioembolic CVA, large vessel atherosclerosis CVA, etc.) demonstrates the highest frequency within the time frame of 06:00 to 12:00. The circadian pattern of CVA is not influenced by prior aspirin consumption. Additionally, the interval between awakening and the onset of CVA was evaluated in 145 patients, revealing that 24 % of ischemic CVA occur within the first hour after awakening.
Pasqualetti et al. [72]	1990	symptom onset time	Any strokes	1, MN	667	193	183	151	140	65	The peaks of CVA occurrence have been observed in the morning hours, during weekends, and in the winter season. Hemorrhagic CVAs do not exhibit circadian rhythmicity; however, they demonstrate weekly and annual patterns. Ischemic CVA displays circadian, weekly, and annual rhythms. No significant differences in CVA incidence have been observed between males and females.
Marler et al. [73]	1989	symptom onset time	Ischemic	2, MN	1075	85	485	320	185	106	During the daytime and early evening (from 8:00 a.m. to 8:00 p.m.), the majority of CVAs in awake patients occurred from 8:00 a.m. to noon, with a decrease observed during the remainder of the day and early evening. The pattern of CVA occurrence in patients receiving aspirin, dipyridamole, or warfarin prior to the CVA remained unchanged, and this pattern was not associated with age, sex, blood pressure at the time of admission, history of hypertension, or the severity of CVA outcomes.
Van der Windt et al. [74]	1988	symptom onset time	Cerebral infarction	1, MN	59	5	22	24	8	7	46 patients (78 percent) experienced cerebrovascular accidents between 6 a.m. and 6 p.m. Only 5 patients (8 percent) suffered CVA between midnight and 6 a.m. Atherothrombotic CVA is primarily not influenced by hemodynamic factors.
Tsementzis et al. [75]	1985	symptom onset time	Any strokes	2, MN	543	83	183	138	139	14	Among 557 patients, 194 experienced subarachnoid hemorrhage, 118 had intracerebral hemorrhage, and 245 presented with thromboembolic CVA. All three types of CVA exhibited the highest incidence between 10:00 a.m. and 12:00 p.m. Intracerebral hemorrhages occurred less frequently between 4:00 a.m. and 6:00 a.m.; however, no significant differences were observed among groups at other times. There was no difference in the onset time of CVA between patients with normal blood pressure and those with hypertension (either treated or untreated). In the intracerebral hemorrhage group, a higher proportion of patients had untreated hypertension. Subarachnoid hemorrhage predominantly occurs during physical activities. Intracerebral hemorrhage is more frequently observed during driving or alcohol consumption. Thromboembolic CVA is more commonly noted during sleep or upon awakening.
Marshall [76]	1977	symptom onset time	Any strokes	6, MN	256	81	69	59	47	0	Ischemic CVA: The highest incidence of CVA occurs during the time period from midnight to 6 a.m. This increase in incidence is statistically significant in both genders (males and females). In males, this increase is observed in both groups of blood pressure (hypertensive and normotensive). In females with diastolic blood pressure less than 110 mmHg, a significant increase at night is noted, although it does not reach statistical significance. Hemorrhagic CVA: No increase in the frequency of CVA is observed during the hours from midnight to 6 a.m. In females, an inverse pattern is observed (a decrease in incidence during these hours). In males, CVA occurs uniformly over a 24-h period, with a slight increase in the evening hours (18:00 to 24:00).
Olivares et al. [77]	1973	symptom onset time	Any strokes	6, MN	127	53	32	16	26	0	The ratio of cerebrovascular accidents due to thrombosis, hemorrhage, and embolism was 6:2:1. No significant difference in the incidence of CVA was observed between males and females. Thrombosis and hemorrhage increased with advancing age, while embolism occurred more frequently in younger patients. Thrombosis typically occurs during periods of rest and in individuals with sedentary occupations. The distribution of weight indicated that obese individuals or those underweight did not have an increased risk for CVA. Hypertension was reported in 62 %, coronary artery disease in 36 %, and diabetes mellitus in 25 % of patients. Other common conditions included smoking and alcoholism. Emotional stress was subjectively assessed by the physician and was present in 12 % of patients with thrombotic CVA, 16 % with hemorrhagic CVA, and 9 % with embolic CVA. Thrombosis, hemorrhage, and embolism were associated with varying temperatures and humidity levels. The peak occurrence of CVA was observed in August and September (the end of the rainy season). CVA risk factors are generally not influenced by ethnic and cultural differences.
Sreekrishnan et al. [78]	2023	symptom onset time	Acute Ischemic	8, MN	1506	360	666	480	0	The highest incidence of cerebrovascular accidents occurred during the daytime (666 cases, 44.2 %), while fewer CVAs were observed at night (360 cases, 23.9 %) and in the evening (480 cases, 31.9 %). The ratio of collateral blood flow status in the evening is higher compared to that during the day and night. Evening CVA may be associated with worse clinical outcomes compared to daytime CVA.	Sreekrishnan et al. [78]
Ignatova et al. [79]	2021	symptom onset time	Any strokes	8, MN	675	81	392	202	0	CVA is classified into three types: ischemic CVA (92 %), hemorrhagic CVA (6 %), and subarachnoid hemorrhage (2 %). No seasonal dependence for the occurrence of CVA has been recorded; however, the highest incidence was observed in June, while the lowest rates were noted in July and October (p < 0.05). A high prevalence of CVA events occurs at the beginning of the week (Tuesday) and at the end of the week (Saturday). The incidence of CVA is highest in the morning and lowest at night.	Ignatova et al. [79]
Author	Year	symptom onset time/ medical presentation time	Type of CVA	Time Blocks (h), starting at	n	23:00–6:59	7:00–14:59	15:00–22:59	Untimed	Findings	Author
Sreekrishnan et al. [78]	2023	symptom onset time	Acute Ischemic	8, MN	1506	360	666	480	0	The highest incidence of cerebrovascular accidents occurred during the daytime (666 cases, 44.2 %), while fewer CVAs were observed at night (360 cases, 23.9 %) and in the evening (480 cases, 31.9 %). The ratio of collateral blood flow status in the evening is higher compared to that during the day and night. Evening CVA may be associated with worse clinical outcomes compared to daytime CVA.	Sreekrishnan et al. [78]
Author	Year	Symptom onset time vs. medical presentation time	Type of Stroke	Time Blocks (h), starting at	n	22:00–05:59	06:00–13:59	14:00–21:59	Untimed	Findings	Author
Ignatova et al. [79]	2021	symptom onset time	Any strokes	8, MN	675	81	392	202	0	CVA is classified into three types: ischemic CVA (92 %), hemorrhagic CVA (6 %), and subarachnoid hemorrhage (2 %). No seasonal dependence for the occurrence of CVA has been recorded; however, the highest incidence was observed in June, while the lowest rates were noted in July and October (p < 0.05). A high prevalence of CVA events occurs at the beginning of the week (Tuesday) and at the end of the week (Saturday). The incidence of CVA is highest in the morning and lowest at night.	Ignatova et al. [79]
Author	Year	symptom onset time/ medical presentation time	Type of Stroke	Time Blocks (h), starting at	n	00:00–07:59	08:00–15:59	16:00–23:59	Untimed	Findings	Author
Zheng et al. [38]	2016	medical presentation time	Intracerebral hemorrhage	8, MN	2904	657	1294	953	0	Patients who experienced intracerebral hemorrhage in the early morning hours (Group 1) or in the afternoon to evening hours (Group 3) exhibited lower coma scores. For Group 1 (00:00–07:59), the odds of a low coma score were 1.72 times greater compared to Group 2 (08:00–15:59), and for Group 3 (16:00–23:59), this odds ratio was 1.95 times greater. No correlation was found between the timing of intracerebral hemorrhage onset and the volume of hemorrhage. The 90-day outcomes for patients (death or major disability) were also unaffected by the timing of intracerebral hemorrhage onset.	Zheng et al. [38]
Korv et al. [17]	2014	symptom onset time	Any strokes	8, MN	8878	1590	4814	2474	0	54 % of CVAs occurred during the daytime. 28 % occurred in the evening and 18 percent occurred at night. Patients experiencing CVA at night were typically younger and predominantly male. The percentage of patients over 80 years old was lowest in nocturnal CVA. Conditions such as atrial fibrillation, heart failure, hypertension, and diabetes mellitus were more commonly observed in evening CVA. Smoking was more prevalent among patients with nocturnal CVA. Treatment times for patients with nocturnal CVA were longer than for others. The timing of CVA onset affects treatment timing; however, therapeutic outcomes and recovery rates were similar across all groups.	Korv et al. [17]

## 3 Results

### 3.1 Study selection

**Fig. 1:**
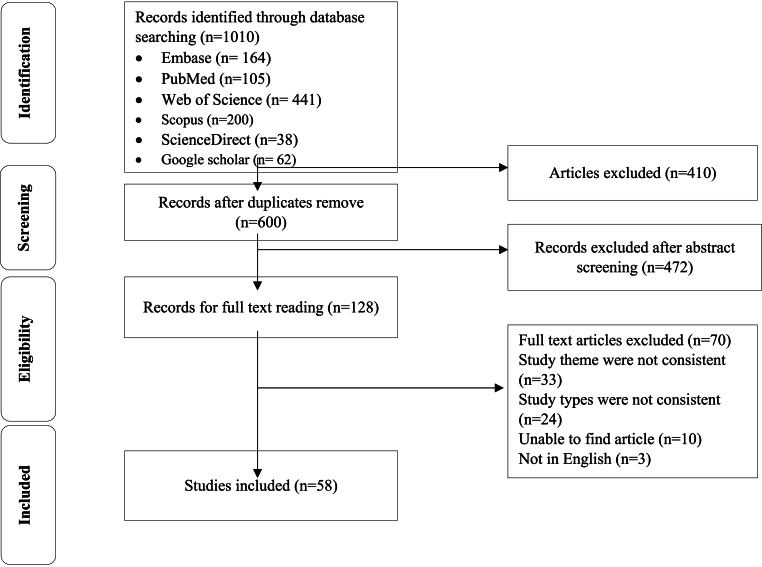
PRISMA flow chart for the study screening process.

Through initial searching, 1010 studies were identified. Following the exclusion of duplicate papers, 600 studies remained. During primary and secondary screenings, 472 studies were also excluded. Finally, 58 eligible articles were included for data extraction (Fig. 1).

The results obtained from data extraction are as follows, and a summary of included article characteristics is presented in Table 1.

The general chronological classification structure (similar in all the studies) was as follows: Night (00:00–05:59), Morning (06:00–11:59), Afternoon (12:00–17:59), and Evening (18:00–23:59).

## 4 Ischemic stroke

### 4.1 Timing of ischemic stroke onset

According to the studies, the occurrence of ischemic stroke happens at specific times of the day. The highest stroke incidence occurred early in the morning hours and decreased during nighttime [21,22]. Kabir et al. reported that 47.8 % of ischemic strokes occurred between 6:00 and 12:00, and there was another peak between 00:00 and 6:00 [21]. These findings are in line with those of Raj et al. [23]. Similarly, Choi et al. also reported that 37 % of ischemic stroke cases occur between 6:01 and 12:00 [22]. Gur et al. and Turin et al. observed that the rate of ischemic stroke incidence is highest in the morning and lowest at night [24,25]. Gupta et al. demonstrated that the circadian distribution of stroke onset remains consistent across age and sex groups, indicating that demographic factors exert a limited effect on this pattern [26].

According to certain studies, the occurrence of ischemic stroke follows a bimodal pattern instead of a single morning peak. Omama et al. demonstrated that strokes follow two differing peaks between 6:00 and 7:59 and another between 18:00 and 19:59 [27]. Similarly, Spengos et al. reported that the highest incidence of stroke occurs between 8:00 and 10:00, with another increase between 16:00 and 20:00 [28]. Certain subtypes of ischemic strokes may evince differences with respect to the occurrence pattern. In their analysis, Naess et al. observed that lacunar strokes occur relatively more frequently at night (from 00:00 to 6:00) compared to other types of stroke [29].

### 4.2 Association between timing of onset and outcomes of ischemic stroke

Evidences indicate that the timing of stroke onset is significantly associated with both severity and prognosis. According to Ryu et al., nocturnal strokes are linked to unfavorable clinical outcomes, including neurological decline and diminished post-stroke functional capacity [30]. Sadhukhan et al. reported that patients with CVA admitted within the nighttime hours (00:00 to 6:00) usually represent a higher National Institutes of Health Stroke Scale (NIHSS) value. The mRNA levels of genes such as BMAL1 and CRY1 were also lower in those experiencing nighttime strokes [31]. However, Wang et al. stated that the proportion of patients with CVA between 00:00 and 6:00 received favorable treatment outcomes (58.6 %) while those with stroke within 18:00 and 24:00 had the lowest proportion for recovery [32].

## 5 Hemorrhagic stroke

### 5.1 Timing of hemorrhagic stroke onset

Several studies approved that there is a specific time period for predominant occurrence of hemorrhagic strokes, whether it is intracerebral hemorrhage (ICH) or subarachnoid hemorrhage (SAH). While numerous studies provided evidence supporting the temporal manifestation of stroke onset, the literature still reflects notable inconsistencies across findings. Some studies report that the peak incidence of stroke occurs during the early morning hours, particularly between 04:00 and 08:00 [33], whereas others indicate a higher occurrence in the evening and nighttime [34].

Studies reporting a pattern of higher incidence of hemorrhagic stroke in the morning are frequently attributed to blood pressure fluctuations and circadian variations in cardiovascular physiology. In this regard, Kabir et al. reported approximately 48.1 % of intracerebral hemorrhage (ICH) cases occurred between 6:00 and 12:00 [21], while Omama et al. observed a peak incidence of hemorrhagic stroke between 6:00 and 7:59, with a secondary peak between 18:00 and 19:59 [27]. Lee et al. observed that the occurrence of subarachnoid hemorrhage has two main peaks: between 8:00 to 12:00 and between 16:00 to 20:00 [35]. However, studies such as Carcel et al. reported different findings; some individuals exhibited a higher incidence of hemorrhagic stroke during evening and late-night hours, particularly between 18:00 to 24:00 [34].

Several investigations emphasized the role of seasonal factors in hemorrhagic stroke incidence. In this context, Kelly-Hayes et al. demonstrated that the peak occurrence of hemorrhagic stroke was observed in winter [36]. These observations corroborate the results of Kocer et al., which documented an elevated incidence of hemorrhagic stroke in winter relative to summer [37].

### 5.2 Association between timing of onset and outcomes of hemorrhagic stroke

The severity and outcomes of hemorrhagic stroke may be related to the timing of onset, although recent studies reported inconsistent findings. Some studies indicated that nighttime strokes are generally more severe. For instance, Zheng et al. report that the patients suffering from intracerebral hemorrhage in the period from 00:00 to 07:59 represent lower coma scores and a significantly higher chance for poor outcomes [38]. In this sense, Wang et al. found no significant differences between three-month mortality rates among patients suffering from strokes at different time intervals [32]. Korv et al. demonstrated that initial stroke severity varies with time of onset, although long-term recovery rates appear similar across onset periods [39].

## 6 Discussion

This systematic review aimed to investigate the relationship between the timing of stroke onset and clinical outcomes in both types of ischemic and hemorrhagic strokes. The study demonstrated that the timing of stroke onset significantly influences both incidence and clinical outcomes. These studies highlighted that the temporal occurrence of ischemic versus hemorrhagic strokes follows markedly different patterns. The high incidence of ischemic stroke occurs early in the morning hours, primarily between 06:00 and 12:00, while some studies identified a bimodal distribution throughout the day. For instance, the evidence on the timing of hemorrhagic stroke events was conflicting; several studies reported a peak incidence in the morning, whereas others indicated a higher occurrence during nighttime. Additionally, stroke onset timing influences the severity of clinical manifestations and treatment outcomes; for example, evidence suggests that nocturnal strokes are associated with poorer outcomes, whereas the strokes occurring in the morning tend to show a more favorable prognosis. These findings can enhance comprehension of stroke temporal patterns and guide the development of preventive measures, timely interventions, and optimized therapeutic strategies.

There are multiple studies indicating that the incidence of ischemic stroke follows a circadian pattern, and the majority occur within the early hours in the morning. For instance, according to Choi et al., Kabir et al., and Raj et al., stroke onset was highly pronounced in 6:00 and 12:00 compared to other time intervals [[21], [22], [23]]. This finding was also observed in studies conducted by Turin et al. and Gur et al. They investigated the relationship of this phenomenon with changes in the physiological status of the body at wake-up time [24,25]. One possible explanation for the observed phenomenon is the daily variation of blood pressure. Blood pressure decreases throughout the night and is followed by a rapid increase after waking up; thus, this sudden change may cause instability of cerebral blood flow, resulting in an increased risk of stroke. Scheer et al. described that an increase in platelet activity and enhanced levels of coagulation factors in the morning hours can increase the probability of thrombosis and vascular occlusion [40].

Nevertheless, certain studies challenged the established temporal pattern; Kelly-Hayes et al. observed that embolic stroke peaks during winter, implying that environmental variables could contribute to the observed temporal variation in stroke occurrence [36]. In contrast, Naess et al. showed that types of CVA like lacunar strokes happen commonly in the night, possibly explaining the different pathophysiological mechanisms behind these subtypes of stroke [29].

Another critical issue is whether the timing of stroke onset influences patient outcomes. Research by Ryu et al. and Sadhukhan et al. indicated that nocturnal strokes are linked to more severe clinical manifestations and worse prognoses. This phenomenon may be attributed to delays in treatment, arising from patients’ inability to detect or respond to symptoms nocturnally, as well as reduced access to transportation and emergency medical services during night hours [30,31]. According to Sadhukhan et al., patients presenting with strokes at night exhibited the highest National Institutes of Health Stroke Scale (NIHSS) at admission, reflecting more severe neurological impairment [31]. Also, Fodor et al. reported that the patients with nighttime strokes rated poorly on cognitive tests such as MMSE during follow-ups, indicating that nighttime strokes can induce an impact on recovery status [41].

Contrary to prior findings, some studies reported no significant association between stroke onset time and patient clinical outcomes. Wang et al. reported no significant difference regarding three-month mortality rates or early reperfusion success among patients with strokes occurring at different times [32]. Such findings point to medical care quality and initial severity of stroke as probably weighted influences in patient outcomes.

From the perspective of biological mechanisms, such as the study of Sadhukhan et al., the genetic impact on stroke outcomes was examined. The present investigation illustrated that expression levels of genes implicated in circadian rhythms (such as BMAL1 and CRY1) are lower in patients with nighttime strokes [31]. These changes can predispose the cases to greater decompensation, cognitive deficits, or depressive episodes, which might compromise the long-term quality of life. On the other hand, Vermeer et al. demonstrated an increase in the risk of an aneurysm rupture during the day and evening, but a lower likelihood for night, which implies a putative protective effect of circadian mechanisms within the body [42].

Zheng et al., reported that the patients experiencing intracerebral hemorrhage (ICH) between midnight and early morning (00:00–07:59) or late afternoon and evening (16:00–23:59) exhibit comparatively lower coma scores. This observation may indicate that intracerebral hemorrhage (ICH) events occurring during these time intervals are associated with greater clinical severity [38]. Similarly, Haapaniemi et al. illustrated that increased risk of ischemic stroke during holidays and weekends may be linked with increased stress, changes in behavior, and even alcohol consumption [43].

However, it is approved that there is no statistically significant difference in the time of stroke occurrence and the mortality or recovery rates in patients. For example, Wang et al. stated that mortality rate at three months after intracerebral hemorrhage (ICH) onset is not associated with the time of stroke [32]. This finding shows that the initial stroke severity, anatomical location of cerebral hemorrhage, and therapeutic interventions may constitute more critical determinants of patient outcomes.

In view of these inconsistent findings, it is concluded that multiple factors contribute to the variability in stroke onset timing and the associated clinical outcomes. A fairly large body of evidence supports the occurrence of stroke during early morning hours and consequent effects on the health outcomes. Nonetheless, several investigations revealed no significant disparities, possibly because such differences were confounded or attenuated by patient-specific factors, variations in healthcare accessibility, or underlying demographic diversity. It is evident that the interaction of physiological, behavioral, and environmental factors plays a pivotal role in this context; thus, further investigations addressing potential confounding variables are warranted to achieve a more comprehensive understanding.

Multiple studies demonstrated that the incidence of hemorrhagic stroke exhibits a circadian variation; however, this rhythmic pattern is not entirely consistent and may differ according to stroke subtype, patient-specific characteristics, and environmental influences. Albarracín et al. and Turin et al. showed that intracerebral hemorrhage (ICH) and subarachnoid hemorrhage (SAH) occurred in the early morning hours, especially in patients diagnosed with these types of conditions [33,44]. This observation aligns with physiological mechanisms (associated with the awakening period), elevations in blood pressure, and alterations in sympathetic nervous system activity. Other studies, such as Carcel et al., had no confirmation of this phenomenon, as the hemorrhagic stroke could occur even in evening hours [34].

A sudden increase in blood pressure upon waking is one of the mechanisms explaining the higher incidence of hemorrhagic stroke in the morning. Typically, blood pressure decreases at night and rises rapidly upon awakening, which can lead to rupture of damaged blood vessels or cerebral aneurysms. Rahman et al. reported that the associated surge in blood pressure, particularly in patients with severe hypertension, is a critical factor in the occurrence of intracerebral hemorrhage (ICH) [45]. However, Kocer et al. indicated that patients with hemorrhagic stroke represent higher blood pressure during nighttime hours compared to other patients. This finding challenges the associated hypothesis and suggests that other mechanisms may also play a role in this process [37]. These findings further imply that the relationship between blood pressure and the timing of hemorrhagic stroke extends beyond a simple circadian pattern; it is likely modulated by additional factors such as individual blood pressure regulation, pharmacological interventions, and sleep-wake states.

Recent studies also concentrated on subgroup variations in the temporal distribution of hemorrhagic stroke onset. Lee et al. and Omama et al. demonstrated that subarachnoid hemorrhage (SAH), in addition to the associated morning peak, also exhibits a secondary peak in the evening hours [27,35]. This finding reveals that the mechanisms causing subarachnoid hemorrhage (SAH) seem different from those of intracerebral hemorrhage (ICH), and factors such as environmental stress, increased physical activity, and hormonal changes in the afternoon could also play some effective roles in this regard. Meanwhile, Vermeer et al. indicated that the avascular rupture of an aneurysm occurs commonly in the afternoon and evening than at night [42]. This phenomenon may reflect the influence of circadian protective mechanisms that contribute to vascular wall stability during nighttime hours.

In the study of Numazaki et al., it was reported that low flow-mediated dilation indicates vascular endothelial dysfunction, followed by the incidence of ischemic stroke in the general Japanese population. This report suggests that flow-mediated dilation can be used as a tool for the identification of the risk of future stroke [80]. Ghaem et al. reported that age >60 years, medical history of hypertension and hyperlipidemia, along with a family history of cardiovascular disease and sudden cardiac death, were associated with myocardial infarction and stroke [81]. Chin-Yu Hsu et al. also stated that the incidence rate of hemorrhagic stroke increases by age, significantly. Although the age-adjusted admission rate of hemorrhagic stroke and mortality after hemorrhagic stroke is decreased, hemorrhagic stroke is still associated with high early and 1-year mortality rates, and women are consistently diagnosed with worse outcomes. Thus, designation of associated strategies to improve the clinical outcomes of these types of patients remains a clinical priority [82].

Application of longitudinal studies to track the CVA patients over time and investigation of the long-term effects of CVA onset timing on clinical and cognitive outcomes are recommended for future research. There is a strict need to understand the relationship between risk factors such as hypertension, coronary artery disease, diabetes mellitus, and sleep patterns with the timing of CVA onset and clinical and cognitive outcomes. The effect of various pharmacological agents (such as antihypertensives and antidiabetic medications) on the timing of CVA onset and therapeutic outcomes is another field of research. The effects of educational programs and public awareness on the reduction of time delay in treatment of patients with CVA and identification of the best procedures for enhancement of public awareness, along with the investigation of cultural influences on CVA-related behaviors (including dietary habits, lifestyle choices, and attitudes toward treatment), are also suggested for future investigations.

### 6.1 Strengths and limitations

Although the present systematic review assessed valid evidence, the included studies covered diverse populations in terms of geography, ethnicity, or socioeconomic status, affecting the generalizability of the results. The principal strength of this study lies in the exhaustive review of all relevant databases and the provision of a complete report of all the evidence of each included article.

## 7 Conclusion

The current research indicated that the timing of stroke occurrence, whether ischemic or hemorrhagic, could influence the incidence and severity as well as the clinical outcome. Some data support that ischemic strokes may occur predominantly in the morning, although other evidence supports that the onset of strokes may show bimodal patterns. With respect to hemorrhagic strokes, while some studies indicated a morning peak, other studies indicate an evening and nighttime peak. In addition, stroke outcome may significantly vary with the time of onset; some publications indicate that strokes at nighttime are more severely affected with poor prognosis. However, other studies found no significant differences regarding long-term outcomes. From this perspective, it is believed that understanding the temporal pattern regarding stroke occurrence and the related association with clinical outcomes could assist in optimizing therapeutic and preventive strategies. A study in this area may elucidate the biological and environmental factors driving the influences on these patterns.

## CRediT authorship contribution statement

**Paria Heidari:** Writing – original draft, Investigation. **Negar Heidari:** Writing – original draft, Investigation. **Habibolah Khazaie:** Writing – original draft, Supervision, Project administration, Investigation, Conceptualization. **Sabra Margaret Abbott:** Writing – original draft, Supervision, Project administration, Investigation, Conceptualization. **Azad Maroufi:** Writing – original draft, Supervision, Investigation, Conceptualization. **Amir Abdolmaleki:** Writing – review & editing, Supervision, Investigation. **Nader Salari:** Writing – original draft, Project administration, Investigation, Conceptualization. **Masoud Mohammadi:** Writing – review & editing, Writing – original draft, Supervision, Software, Project administration, Methodology, Investigation, Conceptualization. **Amir Sharafkhaneh:** Writing – review & editing, Supervision, Project administration, Investigation, Conceptualization.

## Ethics approval and consent to participate

Not applicable.

## Consent for publication

Not applicable.

## Availability of data and materials

Datasets are available through the corresponding author upon reasonable request.

## Funding

Not applicable.

## Competing interests

The authors declare that they have no conflict of interest.

## Abbreviation

CVA: Cerebrovascular accident
PRISMA: The Preferred Reporting Items for Systematic Reviews and Meta-Analyses

## References

[1] Ojaghihaghighi S., Vahdati S.S., Mikaeilpour A., Ramouz A. (2017). Comparison of neurological clinical manifestation in patients with hemorrhagic and ischemic stroke. World journal of emergency medicine.

[2] Temesgen T.G., Teshome B., Njogu P. (2018). Treatment outcomes and associated factors among hospitalized stroke patients at Shashemene Referral Hospital, Ethiopia. Stroke Res. Treat..

[3] Putri I.S., Robbani T.N., Divamillenia D., Pratiwi O.G., I'Tishom R. (2021). A Review.

[4] Owolabi M.O., Sarfo F., Akinyemi R., Gebregziabher M., Akpa O., Akpalu A. (2018). Dominant modifiable risk factors for stroke in Ghana and Nigeria (SIREN): a case-control study. Lancet Global Health.

[5] Ogun S., Ojini F., Ogungbo B., Kolapo K., Danesi M. (2005). Stroke in south west Nigeria: a 10-year review. Stroke.

[6] Janghorbani M., Hu F.B., Willett W.C., Li T.Y., Manson J.E., Logroscino G. (2007). Prospective study of type 1 and type 2 diabetes and risk of stroke subtypes: the Nurses' Health Study. Diabetes Care.

[7] Samuel MB, Aliocha NN, Michel LT, Benjamin LM. Stroke in the Brain Scanner at Kinshasa University Clinics and Marie Biamba Mutombo Hospital: a Case Series Study.

[8] Fodor D.M., Stănescu I.C., Perju-Dumbravă L. (2018). The evolution of disability after ischemic stroke depending on the circadian variation of stroke onset. Balneo Res J.

[9] Koo Y.S., Yu S., Cho K.-H., Jung K.-Y. (2014). Circadian variation of stroke onset and other clinical characteristics: a single-center study. Journal of Korean Sleep Research Society.

[10] Turin T.C., Kita Y., Rumana N., Nakamura Y., Takashima N., Ichikawa M. (2013). Wake-up stroke: incidence, risk factors and outcome of acute stroke during sleep in a Japanese population. Takashima Stroke Registry 1988-2003. Eur. Neurol..

[11] Ripamonti L., Riva R., Maioli F., Zenesini C., Procaccianti G. (2017). Daily variation in the occurrence of different subtypes of stroke. Stroke Res. Treat..

[12] Chen S.-J., Deng Y.-T., Li Y.-Z., Zhang Y.-R., Zhang W., Chen S.-D. (2022). Association of circadian rhythms with brain disorder incidents: a prospective cohort study of 72242 participants. Transl. Psychiatry.

[13] Thomalla G., Boutitie F., Fiebach J.B., Simonsen C.Z., Nighoghossian N., Pedraza S. (2017). Stroke with unknown time of symptom onset: baseline clinical and magnetic resonance imaging data of the first thousand patients in WAKE-UP (efficacy and safety of MRI-based thrombolysis in wake-up stroke: a randomized, doubleblind, placebo-controlled trial). Stroke.

[14] Lee E.-J., Kim S.J., Bae J., Lee E.J., Kwon O.D., Jeong H.-Y. (2021). Impact of onset-to-door time on outcomes and factors associated with late hospital arrival in patients with acute ischemic stroke. PLoS One.

[15] Shokri H.M., El Nahas N.M., Aref H.M., Dawood N.L., Abushady E.M., Abd Eldayem E.H. (2020). Factors related to time of stroke onset versus time of hospital arrival: a SITS registry-based study in an Egyptian stroke center. PLoS One.

[16] Turin T., Kita Y., Rumana N., Nakamura Y., Takashima N., Ichikawa M. (2012). Is there any circadian variation consequence on acute case fatality of stroke? Takashima Stroke Registry, Japan (1990–2003). Acta Neurol. Scand..

[17] Kõrv J., Vibo R., Kadlecová P., Kobayashi A., Czlonkowska A., Brozman M. (2014). Benefit of thrombolysis for stroke is maintained around the clock: results from the SITS‐EAST Registry. Eur. J. Neurol..

[18] Wouters A., Lemmens R., Dupont P., Thijs V. (2014). Wake-up stroke and stroke of unknown onset: a critical review. Front. Neurol..

[19] Elliott W.J. (1998). Circadian variation in the timing of stroke onset: a meta-analysis. Stroke.

[20] Page M.J., McKenzie J.E., Bossuyt P.M., Boutron I., Hoffmann T.C., Mulrow C.D. (2021). The PRISMA 2020 statement: an updated guideline for reporting systematic reviews. Br. Med. J..

[21] Kabir M.R., Hasan A.B.M.K., Hasan M.K. (2019). Circadian variation in stroke: a hospital-based study. International Journal of Advances in Medicine.

[22] Choi Yun Im, Seo Il-Kyo, Kim Doh-Eui, Oh Hyung Geun, Jeong Du Shin, Park Hyung-Kook, Yang Kwang-Ik (2015). Same pattern of circadian variation according to the season in the timing of ischemic stroke onset: preliminary report. Sleep Medicine Research.

[23] Raj K., Bhatia R., Prasad K., Srivastava M.V., Vishnubhatla S., Singh M.B. (2015). Seasonal differences and circadian variation in stroke occurrence and stroke subtypes. J. Stroke Cerebrovasc. Dis..

[24] Gur A.Y., Bornstein N.M. (2000 Mar). Are there any unique epidemiological and vascular risk factors for ischaemic strokes that occur in the morning hours?. Eur. J. Neurol..

[25] Turin T.C., Kita Y., Rumana N., Takashima N., Ichikawa M., Sugihara H., Morita Y., Hirose K., Murakami Y., Miura K., Okayama A., Nakamura Y., Abbott R.D., Ueshima H. (2009). Morning surge in circadian periodicity of ischaemic stroke is independent of conventional risk factor status: findings from the Takashima Stroke Registry 1990-2003. Eur. J. Neurol..

[26] Gupta A.S.H. (2005). Circadian variation in stroke - a prospective hospital-based study. Int. J. Clin. Pract..

[27] Omama S., Yoshida Y., Ogawa A., Onoda T., Okayama A. (2006). Differences in circadian variation of cerebral infarction intracerebral haemorrhage and subarachnoid haemorrhage by situation at onset. J. Neurol. Neurosurg. Psychiatry.

[28] Spengos K., Vemmos K.N., Tsivgoulis G., Synetos A., Zakopoulos N.A., Zis V., Vassilopoulos D. (2003). Two-peak temporal distribution of stroke onset in Greek patients - a hospital-based study. Cerebrovasc. Dis..

[29] Naess H., Idicula T., Brogger J., Waje-Andreassen U., Thomassen L. (2011). High proportion of lacunar strokes at night: the Bergen stroke Study. J. Stroke Cerebrovasc. Dis..

[30] Ryu W.S., Hong K.S., Jeong S.W., Park J.E., Kim B.J., Kim J.T., Lee K.B., Park T.H., Park S.S., Park J.M., Kang K., Cho Y.J., Park H.K., Lee B.C., Yu K.H., Oh M.S., Lee S.J., Kim J.G., Cha J.K., Kim D.H., Lee J., Han M.K., Park M.S., Choi K.H., Lee J., Saver J.L., Lo E.H., Bae H.J., Kim D.E. (2022). Association of ischemic stroke onset time with presenting severity acute progression and long-term outcome: a cohort study. PLoS Med..

[31] Sadhukhan D., Roy A., Banerjee T.K., Krishnan P., Maitra P.S., Mukherjee J., Ghosh K.C., Hui S.P., Biswas A. (2024 Oct 14). Clinical implication of time of ischaemic stroke among post-stroke survivors from Eastern India: a circadian perspective. NeuroMolecular Med..

[32] Wang X., Wang X., Ma J., Jia M., Wu L., Li W., Li C., Wu C., Ren C., Chen X., Zhao W., Ji X. (2022). Association between the time of day at stroke onset and functional outcome of acute ischemic stroke patients treated with endovascular therapy. J. Cerebr. Blood Flow Metabol..

[33] Menéndez Albarracín A., Valls Carbó A., Rabaneda Lombarte N., Yugueros Baena B., Carbonell Gisbert J., Flores-Pina B., Larrañaga De Bofarull M.C., Martínez Sánchez M., Hernández-Pérez M., Bustamante Rangel A., Dorado Bouix L., Gomis Cortina M., Millán Tornè M., Pérez de la Ossa N. (2024). Time of the day and season distribution among stroke code subtypes: differences between ischemic stroke intracranial hemorrhage and stroke mimic. Front. Neurol..

[34] Carcel C.E.-P.R. (2009). Circadian variation of ischemic and hemorrhagic strokes in adults at a tertiary hospital: a retrospective study. J. Neurol. Sci..

[35] Lee J.M., Jung N.Y., Kim M.S., Park E.S., Park J.B., Sim H.B., Lyo I.U., Kwon S.C. (2019). Relationship between circadian variation in ictus of aneurysmal subarachnoid hemorrhage and physical activity. J. Korean Neurosurg. Soc..

[36] Kelly-Hayes M., Wolf P.A., Kase C.S., Brand F.N., McGuirk J.M., D'Agostino R.B. (1995). Temporal patterns of stroke onset: the Framingham study. Stroke.

[37] Kocer A., Ilhan A., Ince N., Bilge C. (2005). The related causes in very early morning onset of stroke. Prog. Neuro Psychopharmacol. Biol. Psychiatr..

[38] Zheng D., Sato S., Cao Y.J., Arima H., Carcel C., Chalmers J., Anderson C.S. (2016). Circadian variation in clinical features and outcome of intracerebral hemorrhage: the INTERACT studies. Chronobiol. Int..

[39] Kõrv J., Vibo R., Kadlecová P., Kobayashi A., Czlonkowska A., Brozman M., Švigelj V., Csiba L., Fekete K., Demarin V., Vilionskis A., Jatuzis D., Krespi Y., Ahmed N., Mikulík R., Safe Implementation of Treatments in Stroke - East Registry Investigators (2014). Benefit of thrombolysis for stroke is maintained around the clock: results from the SITS-EAST Registry. Eur. J. Neurol..

[40] Scheer F.A., Shea S.A. (2014). Human circadian system causes a morning peak in prothrombotic plasminogen activator inhibitor-1 (PAI-1) independent of the sleep/wake cycle. Blood, The Journal of the American Society of Hematology.

[41] Fodor D. (2020). The influence of circadian variation in ischemic stroke onset on the evolution of cognitive status. Balneo Research Journal.

[42] Vermeer S.E., Rinkel G.J., Algra A. (1997). Circadian fluctuations in onset of subarachnoid hemorrhage: new data on aneurysmal and perimesencephalic hemorrhage and a systematic review. Stroke.

[43] Haapaniemi H., Hillbom M., Juvela S. (1996). Weekend and holiday increase in the onset of ischemic stroke in young women. Stroke.

[44] Turin T.C., Kita Y., Rumana N., Takashima N., Ichikawa M., Sugihara H., Morita Y., Hirose K., Murakami Y., Miura K., Okayama A., Nakamura Y., Abbott R.D., Ueshima H. (2010). Diurnal variation in onset of hemorrhagic stroke is independent of risk factor status: Takashima stroke registry. Neuroepidemiology.

[45] Rahman Mahmudur, Rahman Md Matiur, Ahmed Moniruzzaman, Chowdhury Suman Kanti, Alamgir Hossan Md, Hasan Sayful, Islam Nazrul, Rashid Shakila (2024). Circadian variation of stroke in a tertiary level Hospital. Asian Journal of Medicine and Health.

[46] Al-Ahwal Shereen A., Ragab Osama A., Abo Elsafa Ashraf A., Ghali Azza A. (2019). Circadian and circannual patterns of stroke. Egyptian Journal of Neurology Psychiatry and Neurosurgery.

[47] Fodor D.M., Babiciu I., Perju-Dumbrava L. (2014). Circadian variation of stroke onset: a hospital-based Study. Clujul Med..

[48] Hossain Ahmed Manadir (2014). Circadian variation in frequency of stroke in Bangladesh: a Hospital based cross sectional Study. Faridpur Med. Coll. J..

[49] Seo Koo Yong, Yu Sungwook, Cho Kyung-Hee, Jung Ki-Young (2014). Circadian variation of stroke onset and other clinical characteristics: a single-center study. Journal of Korean Sleep Research Society.

[50] Kumar Suresh (2014). Circadian variation in the onset of Acute ischemic stroke. Journal of Rawalpindi Medical College.

[51] Temes R.E., Bleck T., Dugar S., Ouyang B., Mohammad Y., John S., Patel P., Lee V., Prabhakaran S., Quigg M. (2012). Circadian variation in ictus of aneurysmal Subarachnoid hemorrhage. Neurocritical Care.

[52] Yun Sang-Pil, Jung Woo-Sang, Moon Sang-Kwan, Cho Ki-Ho, Kim Young-Suk, Bae Hyung-Sup (2007). Circadian variation of Acute stroke: hospital based Study: hospital based study. 대한한의학회지.

[53] Serena J., Dávalos A., Segura T., Mostacero E., Castillo J. (2003). Stroke on awakening:: looking for a more rational management. Cerebrovasc. Dis..

[54] Bhalla A., Singh R., Sachdev A., D'Cruz S., Duseja A. (2002). Circadian pattern in cerebro vascular disorders. Neurol. India.

[55] Bassetti C.A.M. (1999). Night time versus daytime transient ischaemic attack and ischaemic stroke: a prospective study of 110 patients. J. Neurol. Neurosurg. Psychiatry.

[56] Chaturvedi S., Adams H.P., Woolson R.F. (1998). Circadian variation in ischemic stroke subtypes in the TOAST study. Neurology.

[57] Lago A., Geffner D., Tembl J., Landete L., Valero C., Baquero M. (1998). Circadian variation in acute ischemic stroke: a hospital-based study. Stroke.

[58] Hayashi S., T H., T N., M K. (1996). Daily peaks in the incidence of sudden cardiac death and fatal stroke in Niigata Prefecture. Jpn. Circ. J..

[59] Haapaniemi H. (1992). Early-morning increase in the onset ischemic stroke. Cerebrovasc. Dis..

[60] Roberts M., Opare-Sem O., Acheampong J. (1994). The diurnal variation of stroke in Ghana. Trop. Doct..

[61] Pardiwalla F., Yeolekar M., Bakshi S. (1993). Circadian rhythm in acute stroke. J. Assoc. Phys. India.

[62] Gallerani M., Manfredini R., Ricci L., Cocurullo A., Goldoni C., Bigoni M., Fersini C. (1993 Jun). Chronobiological aspects of acute cerebrovascular diseases. Acta Neurol. Scand..

[63] Wroe S.J., Sandercock P., Bamford J., Dennis M., Slattery J., Warlow C. (1992 Jan 18). Diurnal variation in incidence of stroke: oxfordshire community stroke project. Br. Med. J..

[64] Franke C. (1992). Circadian and seasonal-variation in the incidence of intracerebral hemorrhage. Cerebrovasc. Dis..

[65] Ricci S., Celani M.G., Vitali R., La Rosa F., Righetti E., Duca E. (1992). Diurnal and seasonal variations in the occurrence of stroke: a community-based study. Neuroepidemiology.

[66] Sloan M.A., Price T.R., Foulkes M.A., Marler J.R., Mohr J.P., Hier D.B. (1992). Circadian rhythmicity of stroke onset. Intracerebral and subarachnoid hemorrhage. Stroke.

[67] Toni D., Argentino C., Gentile M., Sacchetti M.L., Girmenia F., Millefiorini E. (1991). Circadian variation in the onset of acute cerebral ischemia: ethiopathogenetic correlates in 80 patients given angiography. Chronobiol. Int..

[68] Herderschee D., Limburg M., Hijdra A., Bollen A., Pluvier J., Te Water W. (1991). Timing of hospital admission in a prospective series of stroke patients. Cerebrovasc. Dis..

[69] Manfredini R., Squarzoni G., Gallerani M., Franceschini F., Bariani L., Fersini C. (1990). Temporal organization of cerebrovascular accidents: a retrospective, preliminary study about stroke in Ferrara, Italy. Rivista Europea per le Scienze Mediche e Farmacologiche= European Review for Medical and Pharmacological Sciences= Revue Europeenne Pour les Sciences Medicales et Pharmacologiques.

[70] Argentino C., Toni D., Rasura M., Violi F., Sacchetti M.L., Allegretta A. (1990). Circadian variation in the frequency of ischemic stroke. Stroke.

[71] Marsh E.E., Biller J., Adams H.P., Marler J.R., Hulbert J.R., Love B.B. (1990). Circadian variation in onset of acute ischemic stroke. Arch. Neurol..

[72] Pasqualetti P., Natali G., Casale R., Colantonio D. (1990). Epidemiological chronorisk of stroke. Acta Neurol. Scand..

[73] Marler J.R., Price T.R., Clark G.L., Muller J.E., Robertson T., Mohr J.P. (1989). Morning increase in onset of ischemic stroke. Stroke.

[74] Van der Windt C., Van Gijn J. (1988). Cerebral infarction does not occur typically at night. J. Neurol. Neurosurg. Psychiatr..

[75] Tsementzis S., Gill J., Hitchcock E., Gill S., Beevers D. (1985). Diurnal variation of and activity during the onset of stroke. Neurosurgery.

[76] Marshall J. (1977). Diurnal variation in occurrence of strokes. Stroke.

[77] Olivares L., Castaneda E., Grife A., Alter M. (1973). Risk factors in stroke: a clinical study in Mexican patients. Stroke.

[78] Sreekrishnan A., Seners P., Yuen N., Olivot J.M., Mlynash M., Lansberg M.G., Heit J.J., Lee S., Michel P., Strambo D., Salerno A., Paredes J.B.E., Carrera E., Albers G.W. (2023 Aug). Elevated Hypoperfusion Intensity Ratio (HIR) observed in patients with a large vessel occlusion (LVO) presenting in the evening. J. Stroke Cerebrovasc. Dis..

[79] Ignatova V.T.L.H.L. (2021). Exogenous temporal factors for stroke onset. Comptes Rendus De L Academie Bulgare Des Sciences.

[80] Numazaki H., Nasu T., Satoh M., Kotozaki Y., Tanno K., Asahi K., Ohmomo H., Shimizu A., Omama S., Morino Y., Sobue K., Sasaki M. (2023 Sep 21). Association between vascular endothelial dysfunction and stroke incidence in the general Japanese population: results from the tohoku medical megabank community-based cohort study. Int J Cardiol Cardiovasc Risk Prev.

[81] Ghaem H., Zibaeenezhad M.J., Sayadi M., Khosravaniardakani S., Parsa N., Razeghian-Jahromi I. (2024 Sep 11). Association of classic cardiovascular risk factors with myocardial infarction and ischemic stroke: a cross sectional analysis of the Shiraz Heart Study. Int J Cardiol Cardiovasc Risk Prev.

[82] Hsu A.C., Vijayarajan V., Cheng Y.Y., Shu M.W.S., Hyun K., Chow V., Brieger D., Kritharides L., Ng A.C.C. (2024 Mar 20). Mortality outcomes in 35,433 patients admitted for acute haemorrhagic stroke in Australia: a population-linkage study. Int J Cardiol Cardiovasc Risk Prev.

